# Hospital accreditation, reimbursement and case mix: links and insights for contractual systems

**DOI:** 10.1186/1472-6963-13-505

**Published:** 2013-12-05

**Authors:** Walid Ammar, Jade Khalife, Fadi El-Jardali, Jenny Romanos, Hilda Harb, Ghassan Hamadeh, Hani Dimassi

**Affiliations:** 1 Ministry of Public Health and Department of Health Management and Policy, Faculty of Health Sciences, American University of Beirut, Beirut, Lebanon; 2 Emergency Social Protection Implementation Support Project, Ministry of Public Health, Beirut, Lebanon; 3 Department of Health Management and Policy, Faculty of Health Sciences, American University of Beirut, Beirut, Lebanon; 4 Department of Information Technology, Ministry of Public Health, Beirut, Lebanon; 5 Department of Statistics, Ministry of Public Health, Beirut, Lebanon; 6 Department of Family Medicine, Faculty of Medicine, American University of Beirut, Beirut, Lebanon; 7 School of Pharmacy, Lebanese American University, Beirut, Lebanon

**Keywords:** Accreditation, Case mix, Healthcare utilization, Contracting, Icd10, Readmission, Lebanon, Payment mechanism, Middle income

## Abstract

**Background:**

Resource consumption is a widely used proxy for severity of illness, and is often measured through a case-mix index (CMI) based on Diagnosis Related Groups (DRGs), which is commonly linked to payment. For countries that do not have DRGs it has been suggested to use CMIs derived from International Classification of Diseases (ICD). Our research objective was to use ICD-derived case-mix to evaluate whether or not the current accreditation-based hospital reimbursement system in Lebanon is appropriate.

**Methods:**

Our study population included medical admissions to 122 hospitals contracted with the Lebanese Ministry of Public Health (MoPH) between June 2011 and May 2012. Applying ICD-derived CMI on principal diagnosis cost (CMI-ICDC) using weighing similar to that used in Medicare DRG CMI, analyses were made by hospital accreditation, ownership and size. We examined two measures of 30-day re-admission rate. Further analysis was done to examine correlation between principal diagnosis CMI and surgical procedure cost CMI (CMI-CPTC), and three proxy measures on surgical complexity, case complexity and surgical proportion.

**Results:**

Hospitals belonging to the highest accreditation category had a higher CMI than others, but no difference was found in CMI among the three other categories. Private hospitals had a higher CMI than public hospitals, and those more than 100 beds had a higher CMI than smaller hospitals. Re-admissions rates were higher in accreditation category C hospitals than category D hospitals. CMI-ICDC was fairly correlated with CMI-CPTC, and somehow correlated with the proposed proxies.

**Conclusions:**

Our results indicate that the current link between accreditation and reimbursement rate is not appropriate, and leads to unfairness and inefficiency in the system. Some proxy measures are correlated with case-mix but are not good substitutes for it. Policy implications of our findings propose the necessity for changing the current reimbursement system by including case mix and outcome indicators in addition to accreditation in hospital contracting. Proxies developed may be used to detect miss-use and provider adverse behavior. Research using ICD-derived case mix is limited and our findings may be useful to inform similar initiatives and other limited-setting countries in the region.

## Background

Resource consumption has been a widely used proxy for severity of illness, and is used by the US Centers for Medicare and Medicaid Services (CMS) and worldwide to develop hospital Case Mix Index (CMI). Case mix has emerged as a reflection of aggregate risk of all individual patients within a hospital, and offers a useful measure to compare hospital performance while using administrative data [1]. CMI is usually derived from Diagnosis Related Groups (DRGs), which were developed in the 1960s by Yale University researchers to evaluate hospital performance using hospital discharges grouped by clinical and resource-utilization similarity [2]. Though DRGs were originally intended as solely a performance measure, they are now widely used for hospital payment mechanisms, increasing transparency, improving efficiency and supporting hospital management [3].

Many countries however do not have a DRG system and rely on International Classification of Diseases (ICD) coding. Yang and Reinke [4] have shown that using an ICD-derived CMI is both a feasible and valid alternative in countries that do not have DRGs. Such findings are not surprising since DRGs themselves are derived from grouping of ICD codes.

The implications of using an ICD-derived CMI are considerable, especially in developing countries where DRG implementation is often more challenging. In Lebanon there has been a past initiative on developing DRGs that remained unavailing due to lack of resources. In such a setting the ability to develop hospital CMI using evidence-based methodology provides valuable knowledge to inform hospital contracting by the largest national insurer which is the Ministry of Public Health (MoPH) [5]. Currently all hospital admissions covered by the MoPH are coded using ICD-10, and as such an ICD-derived CMI would be of greater ease to use due to a decade of experience by both MoPH and hospitals in use of ICD-10.

Since 1962 citizens not covered by any formal insurance schemes are entitled to the coverage of the MoPH. The ministry thus acts as an ‘insurer of last resort’, and has developed its role to lead public health development through a mix of prevention and primary care interventions, and plan and regulate the health sector [6].

The first system for hospital evaluation was developed in 1983, consisting of a classification according to an ‘alpha’ rating which reflected the complexity and quantity of medical services offered, and a ‘star’ rating for hotel/accommodation services of hospitals. The MoPH contracted with all hospitals operating in the country, without any selection mechanism. However, the reimbursement rate was linked to the ‘alpha-star’ category of the hospital. This created a strong financial incentive for hospitals to invest in sophisticated equipment and complex services without rational planning, resulting in increased overall hospitalization cost [7]. Such an unregulated environment resulted in major inefficiencies, especially within the context of the 1974–1990 internal conflict [8]. However the MoPH strategy has since progressed on cautious experimentation and seizing windows of opportunity as they arise [9].

In 2001 the MoPH began the first of two phases in developing an accreditation system [7]. The first was a two-tiered system with basic standards to reflect minimum safety requirements (lacking in the licensing legislation) and accreditation standards to reflect healthcare quality. Indicators used for accreditation emphasized organizational aspects, staff qualification and skills, documented policies and procedures, and data collection on utilization and workload. In this system financial incentives were linked to accreditation category, replacing the previous classification system, and making accreditation a pre-requisite for hospital contracting with the MoPH. Hospitals failing accreditation were no longer eligible for MoPH contracting. At the end of this phase the number of contracted private hospitals decreased from 105 to 85.

The second phase in 2007 expanded the accreditation standards to include performance appraisals, patient care, staff competency testing, appropriateness and implementation of policies and procedures, and additional medical specialties. Concerned over potential loss of contract with the MoPH, hospitals invested remarkable resources and efforts to pass accreditation. This phase saw the number of contracted hospitals increase to 135. Scores were developed for each standard and based on how many standards were fulfilled and at what score, hospitals were grouped into four accreditation categories A to D. The highest reimbursement rate was set for A and B hospitals equally and the lowest for category D. Using D hospitals as reference, reimbursement rate for category C hospitals is on average 15% higher for medical cases and 10% higher for surgical cases. Category A and B hospitals are on average 30% higher for medical cases and 20% higher for surgical cases in comparison to D hospitals. Thus there is a strong financial incentive for hospitals to have higher accreditation category. This system is still currently in use and includes structure, process and to some extent outcome indicators. Such an accreditation-based system thus likely, but not necessarily, reflects better hospital performance in terms of total quality management. Despite the fact that health care providers perceived that hospitals made improvements to quality of care processes through accreditation [10], there is no evidence about the actual impact of accreditation on patient outcomes indicators.

It is important to note that surgical admissions are charged on a flat rate basis while medical admissions are charged according to detailed items used; thus medical admissions also allow a greater likelihood for overutilization and bill inflation. In addition, in order to minimize unnecessary admissions and over-prescription, the MoPH allocates budget ceilings for each hospital based on historical data on service provision. The use of contracting as a ‘soft’ tool for regulation by introducing financing mechanisms such as flat rates and financial ceilings was meant to overcome the prevalent practice of supplier-induced demand in the absence of legislation to allow control of supply.

In the late nineties the reimbursement fees to hospitals were already linked to classification based on structural cost related standards when this evolved from 2001 onwards into an accreditation system with standards based on process and outcome indicators. At the time, the MoPH considered it particularly risky to remove this link, and wanted to keep this financial incentive to create a culture of quality improvement within hospitals and health professionals. The MoPH now intends to re-examine the link between re-imbursement rates and healthcare quality as measured by accreditation, and for this purpose has developed proxy indicators to assess performance and detect provider adverse behavior. Any alternative payment mechanism may have considerable impact on the healthcare system, as it is the major leverage tool that the MoPH can use to drive quality improvement and cost-containment. In addition, currently there is no regular reporting of standardized and comparable hospital-based indicators in Lebanon [11], and the MoPH is interested in using its hospitalization database to develop outcome or proxy indicators.

Our research objective is to evaluate whether or not the current link between hospital accreditation and re-imbursement rate in Lebanon is appropriate, using ICD-derived case mix. We also examine how case mix index and re-admission rate vary between Lebanese hospitals of different characteristics, and the relation between CMI and the proposed proxy indicators.

## Methods

Developing hospital CMI has been primarily done using DRGs, which are essentially ICD codes grouped together according to similar severity and cost. Yang and Reinke [4] have shown that where use of DRGs is not possible, an ICD-derived CMI is a good alternative, especially when using CMI based on ICD costs (CMI-ICDC). We have applied a similar methodology as the US Centers for Medicare and Medicaid Services (CMS) using ICD-10 discharge code costs for all hospitalization admissions covered by the MoPH for the period between June 2011 and May 2012. All other results in this research are also based on hospitalization data for the same period.

The case mix index was developed using the below equation [4]:

$${\text{CMI}}_{h}=\frac{{}_{g}^{I\sum }\left[W_{g}*N_{\mathrm{gh}}\right]/{}_{g}^{\sum }N_{\mathrm{gh}}}{{}_{g}^{I\sum }\left[W_{g}*N_{\mathrm{gn}}\right]/{}_{g}^{\sum }N_{\mathrm{gn}}}$$

Where *h* is the hospital CMI being calculated; W_*g*_ is the weight calculated for each ICD; N_*gh*_ is the number of cases within each ICD in hospital *h*; and N*gn* is the number of cases within each ICD in the total population. Similar to the CMI-ICDC, we applied the same equation to develop a CMI based on surgical procedure cost (CMI-CPTC).

Data was extracted from the ministry hospitalization database using Structured Query Language (SQL) and sorted by case number with patient-level information including ICD-10 discharge diagnosis, age and gender, and hospital-level information on accreditation category, size (total bed capacity) and ownership status (public/private). Permission to use the data in this research was obtained from the MoPH, and all data was anonymized during extraction and thus did not require ethical committee clearance. The total cases extracted were 225,599. Cases with zero recorded costs and those with patient age > 125 years (185 + 132 cases) were regarded as data-entry errors and were removed. We also excluded all cases from 6 specialized hospitals, as well as those from 7 hospitals that had less than 100 admissions each (1268 + 281 cases). Unlike the CMS methodology we choose to not exclude outliers, since CMS does so to reimburse such cases using a separate system, which is not the case of the MoPH. In developing the ICD weights we excluded all ICD codes that had less than 5 admissions in the population, a practice which has been widely used in the literature [12]. A total of 217,550 cases across 122 hospitals (96.4% of all records) were thus included in our study population.

The weight for each ICD code was determined by dividing the average cost of the code by the average cost of all codes. This resulted in 2234 ICD weights in our system, ranging from 0.09-29.9. We applied the weights on all cases admitted to all hospitals, resulting in a hospital CMI for each. The same methodology was repeated for CMI-CPTC. CMI calculations for 50 hospitals were validated manually using raw data from the MoPH database, to ensure proper execution of programming script.

Statistical analysis was conducted using the Statistical Package for the Social Sciences version 16 (SPSS). Descriptive statistics were calculated for the CMI results by each of hospital accreditation, ownership and size. Analysis of variance (ANOVA) was calculated for CMI-ICDC by accreditation and size, and independent t-test used for analysis between public and private hospitals. For ANOVA, Levene’s statistic was used to determine equality of variances, and where this was unequal, Welch’s statistic was interpreted. Where a significant F-ratio was found, Tamhane’s T^2^ post-hoc comparison was done in all cases with unequal variance. Significance level was set at 0.05 for all statistical tests conducted. We examined correlation between CMI-ICDC and CMI-CPTC using spearman’s correlation test. We considered correlation coefficients above 0.8 as good, between 0.8 and 0.5 as fair, and below 0.5 as poor [4]. The same test was also used to examine CMI-ICDC correlation with 30-day re-admission measures and the three proxy measures.

Two measures of 30-day readmission were calculated. The first reflected cases readmitted for the same diagnosis to the same hospital within 30 days of last discharge. The second similarly reflected same discharge readmission within 30 days, but to any hospital, with readmission being calculated for the first admitting hospital. Readmission measures were standardized by sex and age (0-4y, 5-14y, 15-44y, 45-64y, 65-74y, 75-84y, +85y) [13]. All transfer cases and admissions for cancer, mental and behavioral disorders, pregnancy and childbirth were excluded [13-15].

Three proxies currently being considered by the MoPH were examined. These were chosen based on what measures for hospital miss-use or adverse behavior could be developed using the limited data contained within the MoPH administrative database, which the ministry is interested in using for future hospital evaluation. The proxies were developed by MoPH to discourage adverse behavior. The purpose of looking at correlations between these proxies and CMI is to investigate whether or not they are compatible. For example a proxy to discourage misuse by avoiding resource-intensive cases would at least not contradict CMI-based payment. The first proxy examines complexity of surgical admissions. This reflects the proportion of more complex surgical procedures to less complex ones, using admission according to the Common Procedural Terminology (CPT) code with a value above or equal to 100 over those with a value less than 100(CPT K > 100 / CPT K < 100). The second proxy examines the complexity of services for critically ill admissions, and is calculated using the proportion of cases requiring intensive care unit (ICU) admission or ventilator-assisted breathing, to total admissions. This indicator is considered by the MoPH as useful to identify hospitals avoiding critically ill admissions, since they are less financially rewarding relative to other cases. The third proxy examines the proportion of surgical admissions among hospital (medical and surgical) admissions. Since unnecessary admissions are usually medical cases and surgical cases are reimbursed by flat rate, this measure may identify hospitals seeking to maximize profit by over-doctoring.

## Results

### Descriptive statistics

Our study population was composed of 26% accreditation category A hospitals, 7% B hospitals, 46% C and 21% D hospitals (see Table 1). The small number of B hospitals limits the analysis of this category. About 28% of hospitals had less than 50 beds, 43% hade 50 to 100 beds, and 29% had more than 100 beds. The latter two groups admitted an almost equal share of hospitalization cases, with the smallest hospitals admitting less than half of either other sizes. Our sample was comprised of 80% private hospitals and 20% public hospitals.

**Table 1:** **Distribution of hospitals and cases according to hospital characteristics**, **descriptive statistics for hospital CMI based on ICD discharge diagnosis cost** (**CMI**-**ICDC**), **independent t**-**test on ownership**, **and one**-**way between**-**group analysis of variance** (**ANOVA**) **across hospital sizes and accreditation**

		**Hospitals**			**Cases**		
		**n**	**%**		**N**	**%**	
**Accreditation**							
	A	32	26.2%		71,713	33.0%	
	B	8	6.6%		21,646	9.9%	
	C	56	45.9%		93,810	43.1%	
	D	26	21.3%		30,381	14.0%	
**Size**							
	Small (<50 beds)	34	27.9%		41,390	19.0%	
	Medium (50–100 beds)	53	43.4%		89,795	41.3%	
	Large (>100 beds)	35	28.7%		86,365	39.7%	
**Ownership**							
	Public	24	19.7%		66,844	30.7%	
	Private	98	80.3%		150,706	69.3%	
**Total**		122	100.0%		217,550	100.0%	
		**Mean**	**Std. deviation**	**Minimum**	**Maximum**	**P value**	Sig*
**Accreditation**							
	A	1.27	0.24	0.98	1.90	<0.001*	C, D
	B	1.11	0.17	0.88	1.43		
	C	1.09	0.20	0.85	2.15		A
	D	1.04	0.16	0.67	1.45		A
**Size**							
	Small (<50 beds)	1.04	0.16	0.67	1.45	0.001*	Large
	Medium (50–100 beds)	1.12	0.20	0.87	2.15		
	Large (>100 beds)	1.23	0.25	0.88	1.90		Small
**Ownership**							
	Public	1.01	0.10	0.85	1.20	0.003*	
	Private	1.16	0.23	0.67	2.15		
**All**		1.13	0.22	0.67	2.15		

*Significance determined with Tamhane’s T^2^ post-hoc comparison.

### Case mix index

Following the calculation of CMI-ICDC for each of the 122 hospitals in our study population, overall descriptive statistics were calculated, as well as by hospital characteristics. Overall mean CMI-ICDC was 1.13, with hospitals ranging from 0.67 to 2.15. Among accreditation categories mean CMI-ICDC varied from 1.04 to 1.27 with some notable hospital outliers within each category.

A histogram of CMI-ICDC hospital scores revealed a mildly right-skewed distribution, however since this was close to a normal distribution we choose to perform ANOVA to test CMI-ICDC variation by hospital characteristics, while using non-parametric tests (spearman’s correlation) for subsequent analysis. One-way ANOVA was significant for differences by both hospital accreditation and size.

Further analysis of CMI-ICDC revealed that for accreditation categories the only significant difference was category A hospitals having a higher mean CMI (1.27) than both C and D hospitals. Hospitals with more than 100 beds had a significantly higher mean CMI (1.23) than those with less than 50 beds (1.04) hospitals, but not those with 50 to 100 beds (1.12), while the latter two did not differ. To address possibility of confounding due to either volume (number of cases) or hospital size, we performed analysis of covariance (ANCOVA), with our findings regarding accreditation and CMI remaining unchanged. Independent t-test for ownership revealed that private hospitals had a higher mean CMI-ICDC than public hospitals (1.16 and 1.01; 0.003).

In the calculation of CMI-CPTC 115 hospitals were found to have 100 or more cases. A plot of CMI-CPTC and the corresponding CMI-ICDC is shown in appendix figure 2. The correlation between the two measures was apparent, with increasing variability observed at higher values of both CMI results.

Using spearman’s correlation revealed significant correlation between CMI-ICDC and CMI-CPTC at the 0.001 level. This correlation was positive across all hospital characteristics (accreditation, ownership, size) except among hospitals with less than 50 beds and accreditation categories B and D (see Table 2).

**Table 2:** **Spearman**’**s correlations between CMI**-**ICDC and CMI**-**CPTC across hospital characteristics** (**115 hospitals**)

			**CMI-ICDC**	**CMI-CPTC**
**Accreditation**				
	A	R	1.000	0.648
		P		<0.001*
		N	31	31
	B	R	1.000	0.470
		P		0.240
		N	8	8.000
	C	R	1.000	0.571
		P		<0.001*
		N	53	53
	D	R	1.000	0.388
		P		0.067
		N	23	23
**Size**				
	Small (<50 beds)	R	1.000	0.358
		P		0.056
		N	29	29
	Medium (50–100 beds)	R	1.000	0.617
		P		<0.001*
		N	52	52
	Large (>100 beds)	R	1.000	0.704
		P		<0.001*
		N	34	34
**Ownership**				
	Public	R	1.000	0.663
		P		0.001*
		N	22	22
	Private	R	1.000	0.629
		P		<0.001*
		N	93	93
**All**				
		R	1.000	0.655
		P		<0.001*
		N	115	115

* correlation is significant at the 0.01 level.

Examination of correlation between CMI-ICDC and the three proxy measures revealed significant correlation between CMI-ICDC with CPT complexity, ICD/Ventilator use, and surgical proportion (see Table 3). CPT complexity and ICU/Ventilator use are fair proxies for case mix index (CMI-ICDC), but surgical proportion is a poor proxy. Both 30-day readmissions to same hospital and any hospital were poorly correlated with case-mix. Within accreditation categories this correlation held true for CPT complexity in A, B and C hospitals; for ICU/Ventilator use and surgical proportion in A and C hospitals; but not for readmissions.

**Table 3:** **Spearman**’**s correlations between CMI**-**ICDC and other measures** (**122 hospitals**)

	**R**	**P value**	Sig*
**CPT complexity**	0.724	<0.001*	A, B, C
**ICU**/**Ventilator use**	0.521	<0.001*	A, C
**Surgical proportion**	0.404	<0.001*	A, C
**Readmissions (same hospital)**	0.192	0.034*	
**Readmissions (any hospital)**	0.256	0.004*	

*Significance determined with Tamhane’s T^2^ post-hoc comparison.

Individual analysis within each of the 3 proxy measures by accreditation revealed some significant differences.

For CPT complexity the only difference was that A hospitals had more complex surgical procedures than C and D hospitals. Within both ICU/Ventilator use and surgical proportion there was no difference by accreditation. Among the selected measures evaluated, surgical complexity and case complexity are only fair proxies for CMI, while surgical proportion was a poor proxy for CMI.

### Readmission rate

For 30-day readmission measures, the proportion of readmissions increased with increasing accreditation category for both same-hospital readmission ranging from 0.7% in D to 2.7% in A, as well as for any-hospital readmission ranging from 1.3% in D to 4.0% in A (see Table 4). Both readmission measures varied with different hospital sizes, with readmission increasing as hospital size increases. Table 4 also reveals readmission rate differences for both measures, by accreditation and size, but not by ownership.

**Table 4:** **Descriptive statistics for 30**-**day readmissions as proportion of total admissions**, **independent t**-**test on ownership**, **and one**-**way between**-**group analysis of variance** (**ANOVA**) **across hospital sizes and accreditation**

		**30-day readmission to same hospital**				**30-day readmission to any hospital**			
		**Mean**	**Std. deviation**	**P value**	Sig*	**Mean**	**Std. deviation**	**P value**	Sig*
**Accreditation**									
	A	0.027	0.041	0.014*		0.040	0.045	0.001*	D
	B	0.023	0.019			0.025	0.016		
	C	0.015	0.015		D	0.020	0.013		D
	D	0.007	0.006		C	0.013	0.009		A, C
**Size**									
	Small (<50 beds)	0.009	0.010	0.013*	Large	0.014	0.009	<0.001*	Large
	Medium (50–100 beds)	0.016	0.022			0.020	0.019		Large
	Large (>100 beds)	0.027	0.035		Small	0.040	0.040		Small, Medium
**Ownership**									
	Public	0.013	0.011	0.111		0.020	0.011	0.066	
	Private	0.018	0.027			0.025	0.030		
**All**		0.017	0.025			0.024	0.027		

*Significance determined with Tamhane’s T^2^ post-hoc comparison; borderline significance between A and D categories for 30-day readmission to same hospital.

Examining hospital accreditation and readmission measures revealed a hierarchy of means from 0.027 for A hospitals to 0.007 for D hospitals. Significant difference was noted with C hospitals having a higher proportion of readmissions than D hospitals for both readmission measures, and category A having a higher proportion than D for any-hospital readmissions. The difference between category A and D hospitals did approach significance for same-hospital readmissions. More differences were also found with hospital bed capacity, with readmissions being significantly greater in hospitals with more than 100 beds than those with less than 50 beds. For readmissions into any second hospital, those with 50–100 beds also had significantly greater rates than those with less than 50 beds.

## Discussion

Our findings reveal that hospitals in the highest accreditation category have a higher case mix index than those in lower categories. This is not a surprising finding since one would expect a higher accredited hospital, being better resourced, would admit more complex patients and thus have a higher CMI. The literature on the relation of accreditation with case mix index is not well established, in particular considering the differing components of accreditation systems, however increased technical capability has been found to be associated with higher hospital case mix index [16]. However the absence of a difference in CMI between the remaining three accreditation categories suggests that the payment system used by the MoPH which links reimbursement solely to accreditation is not appropriate. The presence of a few hospitals within categories (A and C) with considerably higher CMI than others in the same category implies that hospitals within the same category are not homogenous. Therefore the current system is unfair and induces inefficiency among and within accreditation categories.

One factor that could have especially contributed to the variation seen in hospital CMI is the lack of financial incentives for improving hospital performance. Instead the system has been built to provide hospitals with clear incentives for enhancing healthcare quality in terms of accreditation requirements.

We also found that hospitals with more than 100 beds have a higher CMI than hospitals with less than 50 beds, which may be another reflection of larger hospitals having greater technical capabilities and thus admitting more complex admissions. It is important to note that our findings regarding case mix index and hospital accreditation remained unchanged even after adjusting for hospital size and volume.

The CMI of private hospitals was higher than that of public hospitals, however the range of CMI was wider among private hospitals, with some admitting lower CMI than public hospitals. This suggests that some private hospitals may be shifting resource-intensive patients towards public hospitals, and noting that public hospitals are predominantly in remote areas and most are considered front-line in nature. In the literature case mix index is often variable between private and public hospitals, but tends to be higher in private hospitals, although the opposite may apply depending on the diagnosis groups involved [17,18] Our findings also confirm that, though private hospitals have more advanced structures and technology and can admit higher patient case mix, they are not necessarily more efficient than public hospitals, and confirms the findings of a systemic review on low- and middle-income countries [19].

The correlation between principal diagnosis-based case mix index (CMI-ICDC) and surgical procedure-based case mix index (CMI-CPTC) is overall fair, but it is only among hospitals with more than 100 beds that ICD-CPTC is a good substitute for CMI-ICDC. A principal diagnosis-based CMI can be regarded as a more appropriate measure for hospital CMI, since it considers all admissions to a hospital rather than only surgical ones. A surgical procedure-based CMI favors hospitals with high proportion of surgeries at the expense of those with low proportion. That both CMIs are more closely correlated in larger hospitals is likely due to these hospitals having a larger proportion of their admissions that are surgical cases, and therefore a CMI-CPTC can substitute better for CMI-ICDC. Nevertheless for most hospitals CMI-CPTC would be a less fair assessment of case mix index than CMI-ICDC, and its usefulness is more as a separate additional measure, whereas principal diagnosis-based case mix index is the better measure for the aggregate complexity of patients admitted to a hospital.

Both CPT complexity and ICU/Ventilator use are fair proxies for CMI, suggesting the possibility to use the first two measures as intermediate measures of CMI, or more ideally as separate measures altogether, which could inform contracting as hospital process indicators. The lack of any correlation between surgical proportion and case mix essentially means that a hospital with more surgeries does not translate into a more complex hospital. This may be interesting to explore further, since surgical procedures are less prone to bill inflation and abuse, being based on a flat-rate fee per procedure rather than the open rate of medical admissions. While such cost-containment methods are favorable, it suggests the relation between flat-rate and case mix is a complex one.

In the literature hospital readmissions tend to increase with increasing case mix [20,21]. We used two different measures of 30-day readmissions to capture the varying perspectives of this outcome measure, taking into consideration the local hospitalization practices of hospitals in Lebanon. Both measures yielded similar results, confirming the suggested interpretations. Readmission increased as accreditation category increased, with the lowest accreditation category (D) having lower readmissions than each of category A and C hospitals, though no significant difference was found between these latter two categories. This suggests the relation between readmission and case mix is also a complex one, where readmissions in higher accredited hospitals may be explained by more complex admissions, while readmissions in lower accredited hospitals may rather reflect poorer healthcare quality. In general for both readmission measures hospitals with more than 100 beds had higher readmission than smaller hospitals.

The absence of any difference in readmission between private and public hospitals, even for the any-hospital readmission, suggests that the healthcare quality of public hospitals is not necessarily lower than that of private hospitals, though they are intended as front-line hospitals and expectedly have lower technical capabilities.

It is important to note that caution should always be exercised in linking hospital performance indicators to reimbursement rate. Early evidence from 250 US hospitals showing Pay for Performance (P4P) increased process-quality measures by 3-4% was encouraging and has been used to inform health system reform in the US [22]. However with longer follow up these gains were decreased or lost, and other research has also found P4P to not improve patient outcomes in the long-term [23,24]. More encouraging is recent research from England where P4P in one region resulted in reduction of mortality [25]. As noted by Epstein [26] in the long-term P4P can increase healthcare quality, but the rate at which this occurs depends on the measures and incentives placed.

### Strengths

There is very limited published research from Lebanon and the Middle East – North Africa (MENA) region on reimbursement, case mix and linkages to contractual mechanisms. To our knowledge this is the first time a case mix index is developed for hospitals in Lebanon using an evidence-based methodology. This is also a first for the MoPH in making such an extensive use of its administrative database, and the process involved in implementing this research highlighted several aspects for potential improvement of the database for future use. In addition, the dataset used for all analysis in this research is a very recent one (June 2011-May 2012) and could be used to inform the upcoming MoPH hospital contracting process. The calculation of hospital CMI and ICD weights is relatively easy to perform, and in the case of the latter, more accurate weights for low-volume codes can be obtained as additional cases collected across two or more years.

### Limitations

A system which heavily relies on hospital coding of cases is always prone to coding malpractice [27]. However in our research where current reimbursement is not directly linked to ICD code, there is no clear financial incentive for hospitals to alter coding. The presence of trained MoPH physician controllers that are locally placed at hospitals whose tasks include oversight of codes recorded, as well as proper medical archiving being a criterion for accreditation, would expectedly minimize such miscoding, though some miscoding will continue to occur regardless of control mechanisms. Another limitation is that we have used cost-data in terms of what the MoPH has to reimburse hospitals, and this does not capture the real cost of treating a patient borne by the hospital.

## Conclusion

Recognizing that linking reimbursement to accreditation has contributed to better hospital adherence to the accreditation process, our results support the inappropriateness of basing reimbursement solely on accreditation. It may be explainable that hospitals in Lebanon in the highest accreditation category have a higher re-imbursement rate, whereas it is not justified to have different rates among the three other categories. While some proposed proxies are somehow correlated with case mix index, they are not good substitutes for CMI, and should be used carefully. The main policy implications of our findings are that current hospital re-imbursement based solely on accreditation is not appropriate, and leads to unfairness and inefficiency in the system. Our research suggests that the MoPH should consider associating CMI-ICDC, reflecting hospital case mix, with reimbursement to enhance performance, whereas some proxies developed may be more appropriate to detect miss-use and adverse behavior. In addition, the ministry may consider additional controls on private sector hospitalization together with a scale up strategy for linking hospital performance to reimbursement. Research using ICD-derived case mix index is very limited in particular in the MENA region where this research could prove useful to other countries with similar situations.

## Abbreviations

ANOVA: Analysis of variance; CMI: Case mix index; CMS: US Centers for medicare and medicaid services; CMI-ICDC: Case mix index derived from ICD principal diagnosis cost; CMI-CPTC: Case mix index derived from surgical procedure cost; CPT: Common procedural terminology for surgical cases; DRGs: Diagnosis related groups; ICD: International classification of diseases; ICU: Intensive care unit; MENA: Middle East North Africa Region; MoPH: Ministry of public health; P4P: Pay for performance; SQL: Structured query language.

## Competing interests

The authors declare that they have no competing interests.

## Authors’ contributions

JK and WA conceived and designed the study, and wrote the manuscript. FEJ contributed to the design and revised the manuscript critically for important intellectual content. JK conducted statistical analysis. JR designed programming script for data extraction. HH and HD reviewed statistical analysis and interpretation. All authors participated in the interpretation of the results, and have read and approved the final manuscript.

## Pre-publication history

The pre-publication history for this paper can be accessed here:

http://www.biomedcentral.com/1472-6963/13/505/prepub
